# Adaptors for disorders of the brain? The cancer signaling proteins NEDD9, CASS4, and PTK2B in Alzheimer's disease

**DOI:** 10.18632/oncoscience.64

**Published:** 2014-07-23

**Authors:** Tim N. Beck, Emmanuelle Nicolas, Meghan C. Kopp, Erica A. Golemis

**Affiliations:** ^1^ Developmental Therapeutics Program, Fox Chase Cancer Center, Philadelphia, PA, USA; ^2^ Program in Molecular and Cell Biology and Genetics, Drexel University College of Medicine, Philadelphia, PA, USA

**Keywords:** Alzheimer's disease, Neurodegenerative, Brain, Cancer, NEDD9, HEF1, CASS4, HEPL, PTK2B, PYK2, RAFTK, CAK-β, CAS, Tau, Amyloid, APP, PSEN1, PSEN2

## Abstract

No treatment strategies effectively limit the progression of Alzheimer's disease (AD), a common and debilitating neurodegenerative disorder. The absence of viable treatment options reflects the fact that the pathophysiology and genotypic causes of the disease are not well understood. The advent of genome-wide association studies (GWAS) has made it possible to broadly investigate genotypic alterations driving phenotypic occurrences. Recent studies have associated single nucleotide polymorphisms (SNPs) in two paralogous scaffolding proteins, NEDD9 and CASS4, and the kinase PTK2B, with susceptibility to late-onset AD (LOAD). Intriguingly, NEDD9, CASS4, and PTK2B have been much studied as interacting partners regulating oncogenesis and metastasis, and all three are known to be active in the brain during development and in cancer. However, to date, the majority of studies of these proteins have emphasized their roles in the directly cancer relevant processes of migration and survival signaling. We here discuss evidence for roles of NEDD9, CASS4 and PTK2B in additional processes, including hypoxia, vascular changes, inflammation, microtubule stabilization and calcium signaling, as potentially relevant to the pathogenesis of LOAD. Reciprocally, these functions can better inform our understanding of the action of NEDD9, CASS4 and PTK2B in cancer.

## INTRODUCTION

Roles in cancer and tumor metastasis are well established for the two paralogous scaffolding proteins NEDD9 (neural precursor cell expressed, developmentally down-regulated 9; also known as HEF1 or Cas-L) and CASS4 (Cas scaffolding protein family member 4; also known as HEPL), and for their interacting partner, the kinase PTK2B (protein tyrosine kinase type 2 beta; also known as PYK2, Cak2β, or RAFTK) [1-4]. Based on early identification of these proteins as regulators of integrin-dependent signaling governing survival, proliferation, migration, and invasion, and the recognition of NEDD9 as a major determinant of cancer metastasis [4-8], the majority of subsequent analyses emphasized their cancer-related roles involving these functions. Unexpectedly, recent genetic studies have implicated single-nucleotide polymorphisms (SNPs) in each of these genes in a completely different domain: Alzheimer's disease [9-14]. In part because independent research groups performed these studies, the fact that three closely interacting proteins have all been linked to increased risk of developing Alzheimer's disease has not been appreciated, limiting consideration of relevant mechanisms by which defects in these proteins might affect disease pathogenesis.

The pathophysiology of Alzheimer's disease is known to reflect important contributions from vascular defects, hypoxia and calcium signaling, microtubular integrity, and inflammation. Interestingly, over the past two decades, several studies have identified roles for NEDD9, CASS4, and PTK2B in these processes, but this literature is typically underappreciated in the context of neurodegenerative diseases, in contrast to the focus on this signaling cluster in cancer and metastasis. In this article, we first introduce the pathological features of Alzheimer's disease, and describe recent data linking NEDD9, CASS4, and PTK2B to this syndrome. We then summarize signaling activities of these proteins with particular focus on literature identifying brain or Alzheimer's-relevant functions. Provocatively, these data suggest a new perspective on the breadth of action of NEDD9, CASS4, and PTK2B, extending their roles into the sphere of neurodegenerative maladies. Finally, we discuss how this assembled body of non-canonical functions for these genes increases appreciation of their roles in cancer.

### Pathobiology and genetic basis of Alzheimer's risk

**Figure 1:**
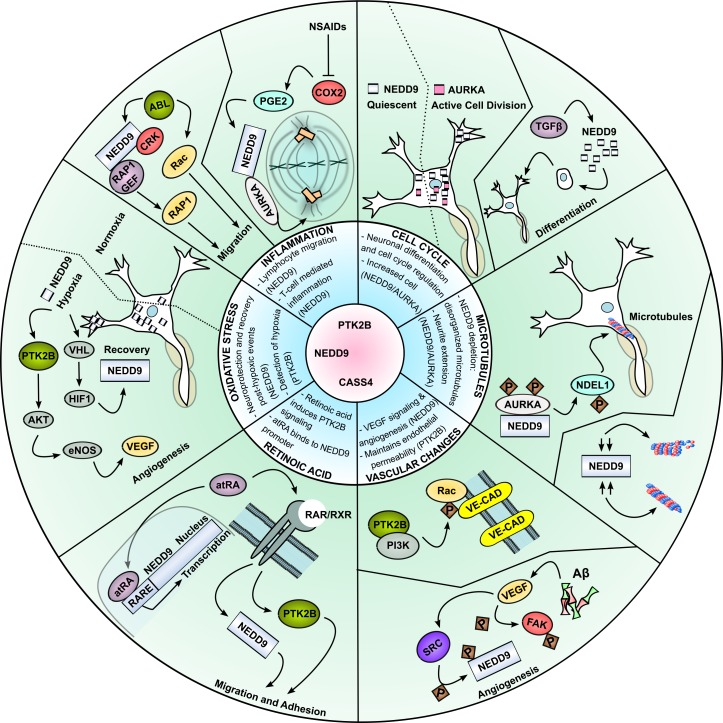
Alzheimer's disease associated processes that involve NEDD9, PTK2B and CASS4 The processes depicted in this figure are: oxidative stress response [115, 116, 118, 121]; inflammatory response (NEDD9 control via PGE2 was described for cancer cells, not neurons) [79, 165, 175]; cell cycle regulation [83, 100]; microtubular alterations [83, 182-184]; vascular alterations [149, 150, 153, 155-157]; and retinoic acid induced signaling and transcription [99, 101, 110, 111]. APP = amyloid precursor protein; Aα = amyloid alpha; R1-R4 = repeat sequences (make up microtubule-binding domain of Tau); RTK = receptor tyrosine kinase.

Alzheimer's disease (AD) is the leading cause of dementia, afflicting over 35 million people worldwide [15, 16]. There are currently no effective treatment strategies for AD, and the disease typically leads to death within 3 to 9 years [15, 17, 18]. Significant structural and functional alterations associated with AD include severe loss of brain volume as well as altered neuronal network activities [17]. β-amyloid peptide (Aβ) plaques, dystrophic neuritis and neurofibrillary tangles composed of hyperphosphorylated tau, are dominant histopathological features [15]. The pathophysiological processes are complex, and include mitochondrial dysfunction (Aβ is a mitochondrial poison [19]), oxidative stress, inflammation, disturbance in cell-cycle re-entry, abnormal cholesterol metabolism, and aberrant vascular changes, with 60-90% of patients with AD presenting with ischemic disease [15, 17, 20-22]. Figure 1 summarizes AD-associated processes relevant to this article.

**Figure 2:**
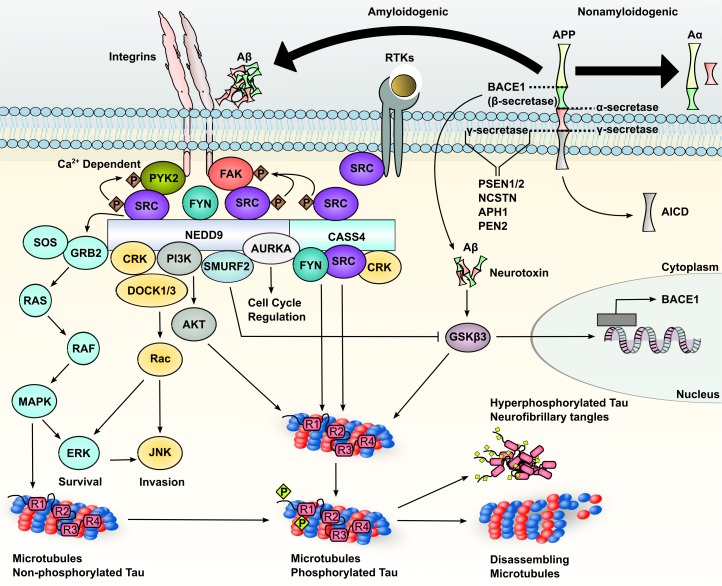
NEDD9, PTK2B and CASS4 as regulators of molecular signaling proposed as relevant in Alzheimer's disease [1, 15, 17, 57, 59, 62, 63, 66, 78, 83, 90, 95-97, 178, 185, 187, 191, 196-198, 200, 226, 227] atRA = all-trans retinoic acid; RAR = retinoic acid receptor; RXR = retinoic X receptors.

Two different subtypes of AD have been identified: early-onset AD (EOAD; <65 years of age) and late-onset AD (LOAD; >65 years old). EOAD accounts for >1-6% of all cases [23] and tends to follow an autosomal dominant inheritance pattern [24]. For EOAD, defects in three genes have been described as causative: mutations, predominantly missense, in amyloid precursor protein (APP), presenilin 1 (PSEN1), or presenilin2 (PSEN2) (Table 1; [24-27]). Presenilin 1 and 2 are critical components of the catalytic site of the γ-secretase complex, a membrane-embedded aspartyl protease required for cleaving the APP C-terminus (Figure 2; [28, 29]).

**Table 1:** Alzheimer's Disease mutations and NEDD9, PTK2B and CASS4 SNPs

Alzheimer's Disease Gene	Association	Genetic Mechanism	Biochemical Phenotype	References
PSEN1	EOAD (30-70%)	Mostly missense mutations (approx. 140)	Reduced γ-secretase proteolytic activity	[28, 224]
PSEN2	EOAD (<5%)	Mostly missense mutations (approx. 10)	Reduced γ-secretase proteolytic activity	[28, 224]
APP	EOAD (10-15%)	Mostly missense mutations (approx. 16)	Increased amount or longer pieces post-cleaving	[224, 225]
APOE	LOAD	ε4 variant	Increased Aβ aggregation and decreased clearance	[33-35]
PTK2B	LOAD	SNP: rs28834970 (Chr. 8)		[14]
CASS4	LOAD	SNP: rs7274581 (Chr. 20)		[14]
NEDD9	LOAD	SNP: rs760678 (Chr. 6)		[9, 13]

The genetic complexity of LOAD is much greater than the relatively well understood genotypes of EOAD. Only apolipoprotein E (APOE) has consistently been identified as an AD susceptibility gene for LOAD, possibly due to the role APOE plays in the clearance of Aβ [16, 30-35]. However, it has been estimated that less than 20% of the risk for the development of LOAD is associated with APOE [36-38]. There has hence been considerable interest in identifying additional contributing factors. Systems biology and large scale genomics studies have begun to provide insights into the underlying multifactorial pathophysiology of AD [14, 32, 39-44], supporting the eventual development of effective treatment strategies. NEDD9, CASS4 and PTK2B have previously been much studied in the context of cancer [1-4]: as summarized below. Their recent identification as candidate regulators of the susceptibility of LOAD suggests numerous specific mechanisms by which they may influence LOAD disease onset and severity (Figure 1).

### Genetic evidence linking NEDD9, CASS4, PTK2B and associated SNPs to neurodegenerative disease

To identify genetic determinants of LOAD, Lambert et al. performed a two-stage meta-analysis of genome-wide association study (GWAS) data. The study analyzed data from 74,046 individuals of diverse ethnicities, and identified a total of 19 AD susceptibility loci, 8 of which had previously been identified. Newly identified loci included SNPs in CASS4 (rs7274581 - chr20: 55018260 T/C) and PTK2B (rs28834970 - chr8: 27195121 T/C) [14]. These had genome-wide significance (*P* < 5 × 10-8) in terms of an association with LOAD [14, 32]. Both rs7274581 and rs28834970 are intronic and distant from splice junctions (2996 and 11923 bases respectively) (allele T|C; http://cadd.gs.washington.edu/score; [45]).

Unlike CASS4 and PTK2B, NEDD9 was not identified as an LOAD associated susceptibility loci by Lambert et al.; in spite of NEDD9 having been implicated as such by several previous studies. The NEDD9 rs760678 SNP was first identified by Li et al. (2008) in a patient population of Caucasian descent, and was proposed to be a common genetic factor in LOAD and Parkinson's disease [10]. In this large-scale association study testing 4692 SNPs, the NEDD9 rs760678 SNP (allele C|G) was identified as the second most significant susceptibility marker (*P* = 0.0051); only rs439401, near APOE on chromosome 19, had a lower p value (*P* = 2.40x10-11; [10]). rs760678 (chr6: 11334654) C/G is located within intron 2, 28266 bases away from a splice site, and has a global minor allele frequency of 0.2984 (http://www.snpedia.com). A potential role for the NEDD9 rs760678 SNP was reiterated by an independent study of genomic DNA from 214 LOAD patients, 135 EOAD patients and 386 healthy individuals. This study found a positive association between rs760678 and LOAD (CC genotype: *P* = 0.016; C allele: *P* = 0.007). No significant difference between EOAD patients and the control group was detected (*p* > 0.1; [12]). Similar observations were made in an additional study with an independent cohort of Han Chinese patients, which compared 383 patients with LOAD with 369 non-affected individuals. A significant difference for both genotype (*P* = 0.003) and allele frequency (*P* = 0.002), between the LOAD group and the control group were determined. Additionally, a significant difference (genotype: *P* = 0.047; allele frequency: *P* = 0.024) between the two groups was maintained even if only subjects without the APOE ε4 allele were considered [13]. However, as mentioned, Lambert et al. did not report a significant occurrence of this particular SNP in their study [14] and Chapuis et al. have described the association between NEDD9 and AD as weak at best [46]. The differences between reports may reflect variation in subject selection, disregard of environmental factors, difference in analysis, or it may simply be due to the underlying complexity of AD. Of note, Chapuis et al. did not indicate in their study if the analyzed cases were cases of LOAD, EOAD or both, perhaps explaining the discordant findings [46]. In 2012, a meta-analysis by Wang et al. concluded that more studies with larger number of samples are required for more precise answers [9-13, 47].

The known biology of NEDD9 closely links its function to CASS4 and PTK2B, as summarized below, additionally suggesting NEDD9 as a compelling target for further studies focused on AD. rs760678 maps near a putative GATA1 transcription factor binding site [10], implying a possible role for the SNP in influencing NEDD9 expression. Intriguingly, the two scaffolding proteins and PTK2B have also been associated with the function of the protein TREM2. TREM2 has been strongly linked to AD and general dementia by several studies [40, 48-50], including the study by Lambert et al., which identified an AD susceptibility SNP (rs9381040; *P* = 6.3 × 10^−7^) only 24 kb away from the 5′ end of TREM2 [14]. Mutations in TREM2 significantly downregulate NEDD9 expression [48] and an established signaling relationship exists between TREM2, its signaling partner DAP12, and PTK2B. TREM2 is a membrane bound protein that is activated by as of yet unidentified ligands to trigger SFK dependent phosphorylation of DAP12, leading to SYK activation, which then engages in a reciprocal activation interplay with PTK2B [51-56]. As we continue to discuss the functionality of NEDD9, CASS4, or PTK2B in relation to LOAD, we view it as most likely that the identified SNPs influence the expression levels of these proteins and thus their functional roles.

### Canonical biology of NEDD9, CASS4, and PTK2B: interactions between these proteins and evidence for action in the brain

Suggestively, NEDD9 and CASS4 are paralogues, with NEDD9 known to interact directly with PTK2B, and CASS4 retaining the motifs required for this interaction. Hence, association of all three with LOAD suggests a common function in the pathobiology of this disease (Figure 2; [1]), paralleling the interactions among these proteins that underlie development and progression of cancer [1-4, 57, 58].

In thinking about this common function, NEDD9 and CASS4 are expressed in many tissues, including the brain [59-61]. NEDD9 and CASS4 are members of the Crk-associated substrate (CAS) family of scaffolding proteins, which also includes BCAR1/p130Cas and embryonal Fyn substrate (EFS; also known as CAS3, CASS3, HEFS and SIN; [1, 2]). CAS proteins share several conserved sequence motifs, including an amino-terminal Src homology 3 (SH3) domain that allows binding with a number of proteins [2], including focal adhesion kinase (FAK) [62] and the FAK paralog PTK2B (Figure 2; [63, 64]). Additionally, CAS proteins contain a “substrate domain”, with multiple tyrosines that form SH2-binding motifs when phosphorylated [1, 4], a 4-helix bundle that provides a docking site for 14-3-3 proteins [65] and GRB2 [66] as well as a C-terminal helical domain that provides another site for interaction with additional proteins, such as SRC family kinases (SFKs), the CAS protein BCAR1 [1], and other signaling adaptors for integrin and receptor tyrosine kinase signaling, such as NSP2/BCAR3/AND-34, SOS and C3G [66-69].

NEDD9 and CASS4 are regulated by and mediate cellular attachment to the extracellular matrix [59, 70, 71]. In studies predominantly performed in cancer cell lines of epithelial and lymphoid origin, following integrin engagement or mechanical stretching, FAK or PTK2B (discussed further below) directly bind and phosphorylate CAS proteins [62], which subsequently leads to further phosphorylation of the CAS proteins by SRC [63, 72-74]. Crk, Crk-L, and BCR/ABL associate with hyperphosphorylated CAS proteins to induce cell spreading (Figure 2; [4, 75]). Reciprocally, overexpression of NEDD9 or CASS4 activates FAK and SRC kinases [3, 59, 76] and organizes complexes that regulate the actin cytoskeleton, contributing to changes in cell adhesion, migration, and invasion, and involve regulation of RAC, DOCK3, and WAVE2 [77]. These changes may vary dependent on cell context, with most studies showing NEDD9 as a positive regulator [70, 78, 79], but some showing a negative role [71, 80, 81].

In addition, in some cell types, NEDD9 can induce the formation of neurite-like cell extensions that depend on an intact microtubule cytoskeleton [82]. Further, NEDD9 associates with and influences the activity of the kinase AURKA to govern appropriate organization of microtubules into a mitotic spindle [83]. Further interactions between NEDD9 and AURKA in G0/G1 cells regulate integrity of the cell cilium [84], a structure important for brain patterning and function [85, 86] that is organized around a microtubule-based axoneme. NEDD9 also associates with the TGFβ effectors and regulatory proteins SMAD3, SMAD6 and SMAD7 [57, 87-89], positioning it to influence cell differentiation status. To date, very few studies have addressed CASS4 function, although roles in control of cell migration have been established: given the high level of domain and sequence conservation between CASS4 and NEDD9 [59], some of the additional roles described above are likely to be relevant.

Protein tyrosine kinase 2 beta (PTK2B) is highly conserved with FAK (90% sequence identity) and together with FAK comprises the complete focal adhesion protein tyrosine kinase family [90]. Intriguingly, PTK2B is expressed with great tissue selectivity, with preferential expression in the central nervous system [91]. PTK2B has a central kinase region flanked by a FERM (band Four point one (4.1), Ezrin, Radixin, Moesin) N-terminal region and a focal adhesion-targeting (FAT) domain at the C-terminal, which allows binding to paxillin [91-93]. Poly-proline sequences on FAK and PTK2B mediate their interaction with the NEDD9 or CASS4 SH3 domain. Auto-phosphorylation of PTK2B induced in response to integrin ligation triggers recruitment of several proteins, most prominently SFKs and GRB2, to initiate migration-inducing signaling [94-96]. In some studies, FAK was shown to oppose the role of PTK2B in inducing reorganization of actin association with focal adhesions and cell rounding, suggesting balanced function of these proteins may be important for tissue organization [97].

Importantly, NEDD9 and PTK2B are highly active in neurologically relevant settings. NEDD9 is expressed in the developing brain [61, 98], and regulates neural crest cell migration [99]. NEDD9 is also required for TGFβ-initiated cell cycle exit and neuronal differentiation in forebrain-derived embryonic progenitor cells [100]. NEDD9 is upregulated in response to retinoic acid in human neuroblastoma cells [101], regulates morphology in glioma and neuroblastoma [80], and has elevated expression and promotes survival and invasion in glioblastomas [78]. Gene expression profiling of the murine striatum after administration of different classes of psychotropics indicated that a cluster of cell cycle regulators, including NEDD9, were significantly enriched [102]; one study has also shown that NEDD9 is upregulated in response to fluoxetine (Prozac), a psychotropic given to some patients with AD [103]. For PTK2B, expression levels are sharply elevated in the front brain shortly after birth [90, 104]. PTK2B localizes to neurites in rat pheochromocytoma cells upon stimulation with nerve growth factor [105], and is a key component of signaling pathways involved in neurite growth and synapse formation [106]. PTK2B and FAK association with adhesion complexes containing both integrins and receptor tyrosine kinases, such as EGFR, to mediate neurite induction by adhesion and growth factors [107]. Further relevant signaling aspects are based on PTK2B-SRC interactions, which couple signals from G-protein coupled receptors (GPCRs) to MAPK activation [96]. Like NEDD9, PTK2B induces the migration and invasion of glioblastoma cells; a role that distinguishes it from its paralog FAK [108].

Lastly, a recent study using a transgenic AD mouse model found that the retinoid X receptor (RXR) agonist bexarotene, used for the treatment of cutaneous T cell lymphoma [109], enhanced clearance of Aβ and improved cognitive function. Multiple studies have described a significant relationship between retinoic acid and NEDD9. The NEDD9 promoter contains binding elements for the retinoic acid receptor (RAR) and RXR (Figure 1; [110]), and NEDD9 is induced by retinoic acid in neural crest cells and neuroblastoma cells [99, 101]. Furthermore, studies of PTK2B in leukemia cells have found it to be upregulated in response to treatment with all-trans-retinoic acid (atRA) [111]. PTK2B signaling, once induced with atRA, was sustained for extended time periods, suggesting a positive feedback loop. Hypothetically, RXR agonists may also increase PTK2B signaling in AD mouse models, and inhibition of PTK2B limit the bexarotene-dependent reduction of Aβ.

### PTK2B and NEDD9 in oxygen responses

In addition to cell-intrinsic defects associated with control of neurite growth and movement, PTK2B, NEDD9, and CASS4 may also influence environmental factors associated with AD. The brain is immensely metabolically active and accounts for roughly 20% of overall oxygen consumption [112]. Even temporary hypoxia can cause permanent damage to neurons [113]. Hypoxia, including particularly chronic hypoxia, has been strongly implicated as causative in neurodegenerative diseases, including AD [114].

While the topic has not yet been significantly addressed for CASS4, Sasaki et al. demonstrated that NEDD9 is highly induced by transient ischemia, and localizes to dendrites and the cytosol of neurons. In this context, NEDD9 helps initiate neural projection formation and may be important for neurite regrowth and tissue recovery following ischemic damage [115]. A study of NEDD9 in colon cancer cells has confirmed that its induction under hypoxic conditions is regulated by hypoxia-inducible factor 1α (HIF1α) and in turn modulates the interaction between HIF1α and its transcriptional coactivator p300 (Figure 1; [116]). While multiple studies find that NEDD9 is upregulated during hypoxia [115-118], one study profiling transcriptomic changes induced by differing states of oxygen tension indicated that a specific set of mRNAs, including NEDD9, is downregulated during hypoxia [119]. The reported discordance may be due to differences in experimental models used (changes in oxygen levels *in vitro* versus induced ischemia *in vivo*) and discrepancies in defining hypoxia (Chadwick et al. state that neurons in culture are typically kept at oxygen levels that are too high to be considered endogenous due to high oxygen consumption in the brain [119]). This topic clearly requires more investigation for definitive conclusions to be made.

Under normoxic conditions, PTK2B is expressed, but typically inactive. However, as shown in a study of rat brains, PTK2B is upregulated and increasingly phosphorylated (i.e., activated) in response to induced hypoxia in microglia [120]. Another study using a mouse hind-limb ischemia model showed that hypoxia acts as a stress signal that is detected by PTK2B, which phosphorylates and activates endothelial Nitric Oxide Synthase (eNOS). eNOS in turn produces nitric oxide (NO), which promotes vasodilatation. During this response, PTK2B also stabilizes AKT kinase activity and intracellular Ca2+ mobilization, which promotes VEGF activity [121, 122]. VEGF helps activate eNOS by triggering the phosphorylation of VEGFR1 and VEGFR2, which leads to PTK2B dependent Ser1176 phosphorylation of eNOS, inducing angiogenesis, migration, and cytoskeletal reorganization [121, 123].

### PTK2B and NEDD9 in the vascular system

Cerebrovascular pathologies are present in 60-90% of patients with AD [124]. Recent studies have linked the function of both PTK2B and NEDD9 to vascular integrity, angiogenesis, and hypertension. Hypertension is of particular interest, for one, because of its general prevalence, and two, because it can be managed pharmacologically and with lifestyle adjustments [125, 126]. Conflicting reports have been published regarding a correlation between hypertension and AD [127-131]; however, postmortem brain analyzes showed significantly less neuritic plaque and neurofibrillary tangle densities in individuals who had received hypertensive medication [132]. To corroborate these findings, in a large study of 2,248 participants, it was determined that use of anti-hypertensives (diuretics, angiotensin-1 receptor blockers and angiotensin-converting enzyme inhibitors) was associated with reduced risk of AD dementia [133].

PTK2B has been linked to key hypertensive events, such as increased cell growth in vascular smooth muscle cells, associated with angiotensin II induced PTK2B phosphorylation [134, 135], and significantly reduced expression of PTK2B in the hypothalamus of mice with established neurogenic hypertension compared to normotensive mice [136]. In a population-based case-control sample study of 2655 individuals, PTK2B variants were also indentified as associated with essential hypertension [137]; although, this observation was not confirmed in a GWAS, in which PTK2B variants were detected, but not seen as significantly associated with hypertension [138]. Strong evidence exists that suggests a direct signaling link between angiotensin, a critical player in the renin-angiotensin system required for blood pressure regulation, vasoconstriction and sodium/potassium regulation [139, 140], and PTK2B. The angiotensin II receptor type 1 transphosphorylates PTK2B to regulate c-Jun expression [141, 142]. Furthermore, PTK2B is critical for angiotensin II-initiated ERK activity [143]. Intriguingly, it has been shown that angiotensin-converting enzyme-1 (ACE1) is elevated in the brains of patients with AD, so are the PTK2B modulators angiotensin II and angiotensin II receptor type 1 [144-146]. Finally, one recent GWAS study has linked NEDD9 to primary open angle glaucoma (POAG), a disease that could be caused by endothelial defects or hypertension. In this case, a NEDD9 intronic SNP was highly associated with POAG (rs11961171, *P* = 8.55E^−13^; [147, 148]). Further study is indicated.

Vascular insufficiency and chronic cerebral hypo-perfusion are clearly involved to some degree in AD, although the cause and effect relationship has not been clearly defined [15, 124]. Aβ induces the potent angiogenic ligand VEGF [149], reportedly activating VEGF-receptors to increase angiogenesis and cell migration [150, 151]. VEGF is a major driver of angiogensis and it has been reported that angiogenesis increases vascular permeability and hypervascularity in AD [152]. NEDD9 and PTK2B have both been linked to angiogenesis and changes in their expression may contribute to hypo-perfusion and vascular permeability in AD. For example, NEDD9 has been defined as critical for a response to VEGF in cancer cells (Figure 1; [150]). When upregulated and activated, NEDD9, along with FAK, is a required target of VEGF signaling in invadopodia, leading to cell spreading and migration [150]. NEDD9 is also a downstream target of PTK2B, important for the regulation of endothelial cell cytoskeletal reorganization necessary for migration during angiogenesis (Figure 1; [3, 153]). Amyloid fibril exposure also increases PTK2B activation in cultured microglia [154]. If similar activation occurs in endothelial cells, PTK2B is positioned to increase endothelial permeability and regulate cell-cell adhesion through its previously described interaction with VE-cadherin (Figure 1; [155- 157]). Work on two transgenic AD mouse models has shown that the VEGFR inhibitor sunitinib re-establishes proper expression levels of proteins involved in vascular integrity, such as Aβ, thrombin, tumor necrosis factor-α, interleukin-1β, interleukin-6, and matrix metalloproteinase 9; NEDD9 and PTK2B were unfortunately not evaluated in this study [158].

### PTK2B and NEDD9 in inflammation

Neuroinflammation has been suggested to play an important role in progression of Alzheimer's disease [33, 159] and epidemiological studies have suggested anti-inflammatory agents may have beneficial effects in AD [160]. Thus far, long term randomized studies have not convincingly shown that anti-inflammatory therapeutics benefit patients with AD; however, the Alzheimer's Disease Anti-inflammatory Prevention Trial (ADAPT), a randomized study of 2528 elderly persons, led to the conclusion that nonsteroidal anti-inflammatory drugs (NSAIDs) have an adverse effect in late stage AD, but reduced the incidence of AD after 2-3 years [161]. ADAPT emphasizes that better understanding of the pathophysiology of AD and the involved inflammatory processes is critical if the inflammatory process is to be effectively targeted with therapeutic intent.

Neuroinflammation involves the activation of microglia, astrocytes, marcrophages and lymphocytes, production of inflammatory cytokines, release of neurotransmitters and production of reactive oxygen species [159]. NEDD9 is important both for lymphocyte signaling [73, 162-164] and lymphocyte migration [79]. NEDD9 has also been implicated in T cell-mediated inflammation, predominantly on the basis of an association with ABL [164-166], with phosphorylation of NEDD9 by ABL and subsequent activation of RAP1 (Figure 1; [165]). PTK2B is required for neutrophil degranulation [167] and inhibition of PTK2B blocks inflammation in murine models of acute lung injury [168] and asthma [169]. PTK2B has also been implicated in the activation of T cells [170, 171] and T cell spreading [172, 173]. In light of the effects of NSAIDs on early stage AD, as reported by the ADAPT study, and the implication of NEDD9 and PTK2B not only in the inflammatory process, but also in AD, additional biological observations regarding NEDD9 are particularly relevant. NSAIDs such as ibuprofen, asprin, naproxen and rofecoxib suppress inflammation by inhibiting cyclooxygenase 2 (COX2) activity [161]. Prostaglandin E2 (PGE2) is a downstream product of COX2 and is therefore suppressed by NSAID treatment [174]. In a first study in colorectal cancer, PGE2 was shown to dramatically upregulate NEDD9 expression [175], raising the possibility that a COX2-PGE2-NEDD9 axis is also involved in AD.

### NEDD9 in microtubule network stabilization

Tau is one of the hallmark proteins of Alzheimer's disease [15], and involved in the pathological formation of tau neurofibrillary tangles [17, 176]. However, tau normally is involved in the assembly and stabilization of microtubules within neurons (Figure 2; [177]), a critical component of proper neuronal functionality [178] and a process disrupted in AD [179-181]. NEDD9 regulation of AURKA activity was first shown to regulate the appropriate organization of microtubules in mitosis [83]. In a particularly exciting finding, AURKA was found to be active in post-mitotic neurons, and to play a significant role in microtubule remodeling during neurite extension. This involves interactions between AURKA, atypical protein kinase C (aPKC), and NDEL1, a partner of the LIS1 gene (mutated in lissencephaly) that regulates dynein. AURKA phosphorylation of NDEL1 was essential to sustain a robust frequency of microtubules from the centrosome, anchoring neurite projection [182-184]. To date, although PTK2B and NEDD9 are abundant in neurites, a PTK2B-NEDD9-AURKA-NDEL1 axis has not been considered in neurite extension or neurodegenerative diseases. Providing additional linkages, glycogen synthase kinase 3 beta (GSK-3β) phosphorylates tau contained in neurofibrillary tangles, and influences the formation of Aβ [185-188]. The NEDD9-regulated kinase AURKA has been shown in cancer cells to phosphorylate GSK-3β, increasing its activity [189]. In some cell types, the E3 ligase SMURF2 negatively regulates GSK-3β [190], but stabilizes NEDD9, promoting AURKA activity [191], raising the possibility of a feedback loop.

Some “canonical” activities of NEDD9 and PTK2B may also influence the formation of tau neurofibrillary tangles as well as APP-associated plaque formation (Figure 2). The SFK FYN binds NEDD9 (and potentially CASS4) [1, 192, 193], and directly regulates the function of PTK2B [194]. Conversely, studies of some CAS family members have shown that CAS-SFK binding is important for activating the SFK [195]. Importantly, FYN is responsible for increased phosphorylation of tau [196-198], a process that induces the dissociation of tau from microtubules, eventually leading to tangles composed of hyper-phosphorylated tau (Figure 2; [15, 199]). In another linkage, some *in vitro* studies have suggested that the activity of Aβ as a neurotoxin requires the induction of α2β1 and αVβ1 integrins, which in turn anomalously activate SFKs and PTK2B, and increase NEDD9 and CASS4 phosphorylation [154, 200].

### NEDD9 and PTK2B in calcium signaling

Defects in calcium signaling have been suggested to play a significant role in the development of AD, and some evidence suggests that Aβ is responsible for disruption of calcium homeostasis [201, 202]. Aβ affects calcium permeability and signaling in brain tissue [203, 204] as well as calcium channel activity [205]. PTK2B is dependent on calcium for activation [105] and modulates NMDA receptor function and MAPK signaling (Figure 2; [104, 206]). NEDD9 activity also regulates and is regulated by calcium signaling. It has been shown in multiple studies that the calcium-regulating hormone calcitonin induces association and phosphorylation of NEDD9, paxillin, FAK, and PTK2B, promoting changes in cellular shape and motility patterns [207-209]. Although this signaling has been predominantly studied in kidney cells and osteoclasts, it is likely relevant in other tissues. In a second connection to calcium signaling control, NEDD9 activation of AURKA supports phosphorylation of the widely expressed polycystin 2 (PC2) calcium channel, to limit the channel's activity and decreases cytoplasmic calcium. Reciprocally, calcium induction of calmodulin (CaM) binding to AURKA supports the AURKA and NEDD9 interaction [210-212]. Relevant connections between calcium homeostasis and NEDD9 and PTK2B are hence clearly established and it would likely be of immense value to investigate the functional impact of this interplay in the context of AD.

### Conclusions, and return to cancer biology

Alzheimer's disease, and particularly LOAD, is immensely complex, and our understanding of this disease on a molecular level is still limited. While genetic analyses to date imply heterogeneous origins for LOAD, the fact that three genes with close functional relatedness have separately been identified as contributing to this disease is highly suggestive of an important conserved pathway. For historical reasons, the bulk of molecular characterizations of NEDD9, CASS4, and PTK2B have stressed their roles in cancer. However, the data summarized above provide a roadmap for systematic investigation of these proteins in the regulation of hypertension, angiogenesis, hypoxia, calcium signaling, inflammatory response, and microtubule dynamics: all phenotypes highly relevant to LOAD. Given the most likely function of the SNPs is to activate or depress signaling of the NEDD9-CASS4-PTK2B axis, their action in LOAD may involve simultaneous subtle regulation of all these phenotypes, with cumulative effects in aged individuals.

Finally, while the main focus of this review has been on the roles of NEDD9, CASS4, and PTK2B in LOAD, many of the LOAD-relevant processes can further highlight the significance of these proteins in cancer. In cancer, discussion of action of the NEDD9-CASS4-PTK2B scaffolding and signaling axis almost invariably focuses on the regulation of cell migration and invasion, following the upregulation of NEDD9 and activation of PTK2B in many tumors. Of note from a therapeutic standpoint, inhibitors of FAK are in clinical development [213-216] and these inhibitors are also active against PTK2B [214]. The data reviewed above suggest that use of these inhibitors may have significant side effects impinging on function of the vascular and immune systems. At this point, whether such effects would be beneficial or negative is difficult to predict; however, we suggest that accumulated data now indicate that specific analysis of this question is worthy of preclinical investigation.

These data also suggest additional ways of thinking about NEDD9, PTK2B, and CASS4 in tumor cells. For example, renal cell carcinoma (RCC) is almost entirely dependent on loss of VHL and induced hypoxic signaling involving VEGFR [217-219]. FAK/PTK2B inhibitors may be expected to behave differently in this class of tumors than others with less dependence on this signaling pathway. We believe that in order to advance cancer therapy it is important to target signaling nodes, functions, and malignant cellular reprogramming in order to maximize prevention, treatment and diagnosis [220-223]. Understanding the roles of scaffolding proteins is important in this regard, as these proteins often interconnect signaling cascades and phenotypic revulators. The described collection of functional relationships for NEDD9, CASS4 and PTK2B in AD is a generalizable approach of thinking about genotypes, phenotypes and ultimately functionality to understand diseases ranging from Alzheimer's to cancer.
